# P-17. Worldwide Impact of Increased Recombinant Zoster Vaccine Coverage on the Burden of Herpes Zoster, based on 11-year Clinical Trial Follow-up Data, in Adults Aged ≥50 Years

**DOI:** 10.1093/ofid/ofae631.225

**Published:** 2025-01-29

**Authors:** Desmond Curran, Sean Matthews, Nikolaos Giannelos

**Affiliations:** GSK, Wavre, Vlaams-Brabant, Belgium; GSK, Wavre, Vlaams-Brabant, Belgium; GSK, Wavre, Vlaams-Brabant, Belgium

## Abstract

**Background:**

Herpes zoster (HZ) is a viral disease impacting patients quality of life due to pain and rash. It is a vaccine-preventable disease with approximately 15 million cases observed annually in individuals ≥50 years of age (YOA) worldwide. Recombinant zoster vaccine (RZV) has proven effective in protecting against HZ infection. This is the first study evaluating the potential increased (incremental) public health benefits - in terms of HZ cases averted - of vaccinating adults ≥50 YOA worldwide with RZV.

**Table 1:**
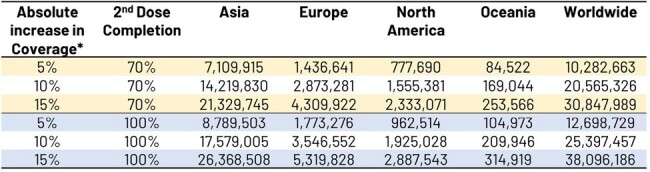
Number of Herpes Zoster cases avoided by region as a function of increasing vaccine coverage and second dose completion levels *Incremental coverage: impact observed due to increased coverage of X%

**Methods:**

A previously published static multi-cohort Markov model with an annual cycle length, and a lifetime horizon was used, which incorporated the following inputs : population estimates, and age-gender specific mortality rates from the latest (2022) United Nations data, depicting populations on December 31, 2023; HZ incidence rates from a recent (2021) meta-regression analysis of HZ burden across the globe (Asia, Europe, Northern America, and Oceania); RZV efficacy modelled based on 11-year clinical trial follow-up data [NCT02723773].

**Table 2:**
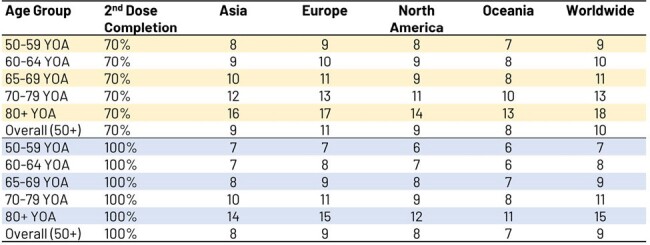
Number needed to vaccinate to prevent Herpes Zoster cases by age and region as a function of increasing vaccine second dose completion levels YOA, years of age.

**Results:**

Assuming a 70% second-dose completion rate in the general population ≥50 YOA worldwide, absolute increases in uptake of RZV by 5% would reduce the number of expected cases by >10 million over the remaining lifetime of vaccinated individuals (see Table 1). Numbers needed to vaccinate (NNV) to avert 1 HZ case by age group worldwide varied from 9 in 50-59 YOA to 18 in ≥80 YOA, and 10 overall in ≥50 YOA (see Table 2). Variations observed by region and age reflected varying inputs, i.e. population demographics, HZ incidence rates, life expectancy and vaccine efficacy.

**Conclusion:**

Even with a relatively modest increase of coverage rates worldwide, RZV is projected to prevent millions of HZ cases due to the long term protection it affords. Lower NNVs are observed in younger vaccinated cohorts, irrespective of region, outlining the relative merits of age of vaccination.

Funding: GSK

**Disclosures:**

**Desmond Curran, PhD**, GSK: employee|GSK: Stocks/Bonds (Public Company) **Sean Matthews, MSc**, GSK: Advisor/Consultant **Nikolaos Giannelos, PhD**, GSK: Employee|GSK: Stocks/Bonds (Private Company)

